# Towards developing and validating Quality Physical Education in schools—The Asian physical education professionals’ voice

**DOI:** 10.1371/journal.pone.0218158

**Published:** 2019-08-01

**Authors:** Walter King Yan Ho, Md. Dilsad Ahmed, Selina Khoo, Chee Hian Tan, Mitra Rouhi Dehkordi, Mila Gallardo, Kicheon Lee, Yasuo Yamaguchi, Yuping Tao, Chunong Shu

**Affiliations:** 1 Faculty of Education, University of Macau, Taipa, Macau; 2 Faculty of Education, Department of Elementary Education, University of Alberta, Edmonton, Canada; 3 Sports Centre, University of Malaya, Kuala Lumpur, Malaysia; 4 Faculty of Sports Science and Recreation, Universiti Teknologi MARA, Malaysia; 5 Faculty of Physical Education, Farhangian University, Tehran, Iran; 6 College of Sports, Physical Education and Recreation, Mindanao State University, Mawari, Phillipines; 7 Department of Leisure and Sports Studies, Korea University, Seoul, South Korea; 8 Department of Physical Education, Kobe University, Kobe, Japan; 9 Chengdu Sport University, Chengdu, China; 10 Hunan Normal University, Hunan, China; TNO, NETHERLANDS

## Abstract

Physical education professionals aim to develop quality programmes for physical education. This study aimed to develop and validate a scale using professionals’ perceptions of Quality Physical Education *QPE* in Asia using twenty-four items regarding QPE quality issues. The items covered status and roles, development of educational elements and supportive features in physical education. A sample of N = 799 sport and physical education professionals from eleven Asian cities participated in this questionnaire survey. Twenty-four items relating to QPE were examined via exploratory factor analysis (EFA) using maximum likelihood extraction and direct oblimin rotation methods. Nevertheless, only 20 items were extracted following the EFA examination. Items 1, 9, 14 and 18 were excluded because of low factor loadings. The remaining items were clustered into four subscales: Development and Supportive Elements for Quality Physical Education in Schools (DSFQPE; α = .918), Core Values of Quality Physical Education (CVPE; α = .908), Curriculum Arrangement of Physical Activities (CAPA; α = .884) and Provision and Norms in Physical Education (PNPE; α = .865). The Cronbach’s alpha coefficient (α = .875) indicated excellent internal consistency for the overall measure. Furthermore, the 4 retained factors from the EFA were assessed via robust confirmatory factor analysis (CFA). The 4-factor model demonstrated a good fit with the data (CMIN/DF = 3.450, CFI = .928, TLI = .916, PCFI = .801, RMSEA = .078). The study identified a 4-factor structure with internal consistency and acceptable interfactor correlations. The structure seemed to be applicable, including the twenty items identified as useful and necessary tools for the framework of analysis in the investigation of diverse settings for the study of quality physical education.

## Introduction

There has been worldwide concern about ensuring the quality development of physical education in schools [1–2]. The origins of this debate date back to the work of UNESCO in 1978 when the organization initiated the proposal of an International Charter on Physical Education and Sport. Past discussions on this agenda have widened our understanding, and the present insufficiency in handling the matter has captured scholarly attention. For example, the National Association of Sport and Physical Education (NASPE) (2004) [3] listed the areas of curriculum, instruction, assessment, academic learning time and the improvement of supporting aspects, such as facilities, resources, and professional education, as the areas of highest concern. The connections of these educational aspects to attaining quality output in physical education have been examined, as active lifestyle development, students’ health improvement, and students’ quality of growth in aspects such as values and attitudes in sport and physical activities, and habits for regular exercise, along with concerns regarding the efficiency of physical education in meeting challenges, such as gender issues, inclusive education, racial challenges, and constraints from religious, traditional and cultural practices cannot be addressed only by reforms in curriculum or the introduction of innovative instruction and assessment in learning. Such understanding turned out to be the core agenda set by sport and physical education professionals at the UNESCO meeting for Quality Physical Education at Porto Novo in 2005.

The difficulties in the development of quality physical education due to the interwoven relationship of various developmental tasks have been investigated. When nations have attempted reform work in physical education, the environmental, cultural and economic conditions and the educational background have served both as facilitators and also barriers, which, in some cases, have made development difficult. The observation of this difficulty traces back to the works of while observing the educational development in developing countries. In his report, he emphasized that for a reform to be functionally suitable to a nation, the readiness of the educational field was critical. Thus, the question arises as to what issues of concern are perceived as important in the development of quality physical education. The understanding of this issue may help to foster the establishment of a comprehensive framework for research and development [4]. The current research expected to arrive at this understanding while learning about what has been achieved and is perceived as important by sport and physical education professionals in Asia.

## Current research

NASPE (2004)’s standards on Quality Physical Education highlighted the concerns of effective development in curriculum, instruction, assessment, academic learning time and the improvement of supporting aspects, such as facilities, resources, and professional education. The recent debate included the agendas of other aspects in achieving the organization’s goal. For example, in Singapore, the desire to improve the quality development of physical education limited the identification of solutions to staffing issues, the inadequate duration of physical education lessons and class size [5]. In China, quality-improving approaches in physical education have become a mere fantasy, as it is common to have 50 to 60 students in a single class, and 80 students is the norm [6]. The lack of adequate space and equipment in physical education made quality improvements difficult. In Bahrain, traditional barriers and parental disapproval served to discourage girls from participating in physical education lessons, and in the Taiwan region, the cultural bias, facilities, equipment and resources posed challenges to the development of physical education [7]. Sarwar et al. (2010) [8] discussed the development of physical education in the industrial city of Gujranwala in Pakistan, where the major problems regarding physical education development included the lack of funds, space, and facilities and the lack of interest from staff, students and parents. The World Bank 2014 [9] report on educational development in South Asia appeared to provide an understanding of the situation. This report indicated that primary schools were almost fully funded but also warned about the importance of needing to do more to improve the quality of education.

The debate on Quality Physical Education has seemed to indicate two camps, with one focusing on educational matters and the other focusing on supportive aspects. These two perspectives, although acting independently, are inter-connected. Their intimate relationship forms the guidelines in most of the reforms in physical education. For example, the 2000 educational reform in Hong Kong made suggestions on curriculum arrangement, but much of the discussion focused on the holistic plan to reach the goal of generic skills development and learning to learn. The introduction of integrated concepts in subject learning, career development, co-curricular concepts and seed projects in Learning to Learn formed the initiatives to identify the inter-connected knowledge between curriculum, instruction and information on environmental needs, facilities and even policies in gender and equality arrangements [10].

For example, McNeill, Lim, Wang, Tan and MacPhail (2009) [5] discussed Quality Physical Education in Singapore, and highlighted class size, curriculum time and professionals’ qualifications as the main constraints to the development of Quality Physical Education programmes in schools. That document highlighted the need for a holistic understanding of factors including class arrangement, opportunities in physical activities, support for teachers, schools and parents and provision of facilities, equipment and venues in addition to those concerns about content knowledge, instructional methods and assessment. Although Hardman (2009) [11] investigated the differences between countries regarding curriculum design, the status of PE in primary and secondary schools, instructional time allocation, and general practices in physical education, he made no attempt to determine the actual concerns of professionals regarding Quality Physical Education. His research focused mainly on the gap between ‘promise and the reality’. However, the measuring technique used in reporting the cases was means, standard deviations and frequencies (percentages) only, instead of a validated scale.

These examples from Singapore and Hong Kong SAR indicated the need to adopt holistic concepts for the development of Quality Physical Education. A regular, quality programme for physical education in schools is necessary, but evidence has also indicated that pupils are more likely to be physically active when a well-established school environment is in place [12]. The achievement of quality growth in physical education requires innovative thought on various aspects, both educational and practical, that address issues such as improving venues, facilities and equipment planning and the management of proper policies to ensure equal and extended opportunities in learning. In response to this challenge, the co-curricular concept for school leisure activities or after-school programmes in Macau SAR and Singapore are the latest attempts to extend learning opportunities through the holistic perspective [13–14].

To our knowledge, a psychometrically sound instrument does not exist to assess professionals’ perceptions of Quality Physical Education. The International Council for Sports Pedagogy recognized that without knowing the concerns of professionals, it would be difficult to identify a proper focus for developing Quality Physical Education in schools. Knowing professionals’ perceptions would be productive because they are educated, have earned qualifications in the same/related fields, have an understanding of the profession and are the first to deal with the policy implemented by the government. Therefore, in 2011, the four member associations (The International Society for Comparative Physical Education and Sport ISCPES, the International Association of Physical Education for Girls and Women IAPESGW, and the Federation Internationale D’education Phisique FIEP) of the International Committee of Sport Pedagogy (ICSP) (a working group of the International Council of Sport Science and Physical Education) initiated a collaborative project to envisage the issues and framework for Quality Physical Education programmes [15].

The subject of physical education exists, more or less, in all Asian countries. However, none of the countries in this region properly follow the UNESCO guidelines for Quality Physical Education. For example, in India, physical education is not yet considered as a compulsory subject, while Macau and Hong Kong have structured curricula but still have several issues such as funding, time allocation, staffing, etc. In the Philippines and Iran, inadequate facilities, low salaries, and social issues are some of the crucial issues. In China and Japan, inadequate government administration, time for PE, social recognition of PE teachers, lack of students’ understanding of the importance of physical education classes, curriculum and assessment of teaching staff, etc., are some of the barriers to the PE profession [16–18]. Similarly, all cities included in this research have their own problems and issues in running the curricula smoothly. The status of physical education in Europe and in many American countries is quite stable in comparison to Asian countries, as they have adequate time allocation for PE classes [19]. In contrast, the USA faces the problems of large class size, cuts to teaching staff and PE programmes every year, budget deficits, etc. [20]. As in other areas, in Africa, shortage of facilities and trained personnel, including inadequate supervision, are serious problems. In some of the Central and Latin American countries, including countries in the Caribbean, involving students in PE is considered a waste of time, and in other places, PE is considered merely as leisure time. The literature reviews on the status of physical education on different continents offers a mixed message. Therefore, the researchers of this study sought to construct a tool to implement with professionals in Asian cities to help measure the perception of professionals from other continents. In other words, valid and reliable measures of perceived Quality Physical Education are required to determine whether Quality Physical Education is achieved in the schools of various countries. This understanding could further provide for a comprehensive and productive programme to promote student involvement and increase student gains from their structured physical education programmes. Consequently, the question for this research is as follows: what are the factors that underpin professionals’ perceptions of Quality Physical Education in Asian countries? To answer this question, the overarching aim of the study is to develop a valid and reliable tool to investigate what has been achieved and professionals’ perceptions regarding Quality Physical Education in school settings. The investigation intends to uncover how Quality Physical Education is best understood and practiced by professionals and to develop a basic framework for the investigation of Quality Physical Education in schools in Asia. Quality Physical Education is defined as a planned, progressive, inclusive learning experience that forms part of the curriculum in early years, primary and secondary education. In this respect, Quality Physical Education acts as the foundation for a lifelong engagement in physical activity and sport. The learning experience offered to children and young people through physical education should be developmentally appropriate to help them acquire the psychomotor skills, cognitive understanding, and social and emotional skills they need to lead a physically active life (Source: adapted from Association for Physical Education (afPE) Health Position Paper, 2008) [21–22].

## Methods

A questionnaire was developed as a strategy for data collection. Physical education teachers and sport professionals from schools and universities were invited to participate in the study. Seven hundred ninety-nine professionals from 11 Asian cities participated in this study (Tables 1 and 2).

**Table 1:** Number of participants in the QPE survey in Asia.

COUNTRY	Cities	Primary School PE Teacher	Secondary School PE Teacher	Teacher in Universities	Total
China	Macau SAR	18	18	24	60
China	Taipei	16	66	17	99
China	Changsha	7	25	58	90
China	Chengdu	24	21	40	85
India	Amravati	20	26	37	83
Iran	Teheran	20	20	40	80
Israel	Tel Aviv	4	3	10	17
Japan	Kobe	27	20	40	87
Korea	Seoul	15	20	39	74
Malaysia	Kuala Lumpur	17	20	46	83
Philippines	Mawari	7	7	27	41
Total	11	175	246	378	799

*Note*: N = 399 used for EFA + N = 400 used for CFA = 799 Total

**Table 2:** Number of participants by gender and professional status.

	Professional Status	N
Male, N = 500	Primary School PE Teacher	105
	Secondary School PE Teacher	145
	University Teachers	**250**
	Total	500
Female, N = 299	Primary School PE Teacher	50
	Secondary School PE Teacher	104
	University Teachers	145
	Total	**299**

Ethical permission has been obtained to conduct this current study from the University of Macau (Research and Development committee). After ethical approval was granted by the University of Macau (first author’s institution), the Principal Investigator (PI) discussed the methodology and aims of the study with the co-authors and colleagues. The co-authors subsequently discussed the research work with their own university to receive permission, as well as with other universities/schools in their respective cities to collect data from the identified professionals in the beginning of 2013. The process of data collection lasted for thirteen months (January 2013 to February 2014). The data collection included information sheets for the participants, consent forms and the questionnaire. All subjects participated in the study voluntarily. The PI also discussed the project in detail with professionals during conferences prior to data collection. The participants were asked to return the questionnaires directly to the researchers using the envelopes provided by the research team or by personally giving them to the researcher in their own city. Furthermore, it was ascertained that all included participants had physical education certifications and were also pursuing a career in physical education.

The current study consisted of two stages, the ‘First Stage’ and the ‘Second Stage’. The first stage included the development of the questionnaire/dimensions/face & content validity [15, 23–24]. The second stage involved having participants respond to the questionnaire and using these data to establish the factors of Quality Physical Education programmes [15, 23–24].

## Questionnaire language

Participants completed the measures in English. Although the study participants included only PE professionals working in educational institutions (primary and secondary schools and universities), the items in the questionnaire were written in simple English; therefore, it was assumed that the participants would not find it difficult to understand the exact meaning of the questions. For example, it has been noticed that some countries, such as India, Malaysia, and Israel, have a British colonial heritage that impacts English language knowledge, while others, including the Philippines, have significant American influence. In addition, in the other participating countries, such as Iran and Korea, people understand English at a high level, and a substantial proportion of the population from all ethnic backgrounds speak English well, even if it is not their “native” language.

However, there was a concern with Japan, Korea, and China. To ensure that participants’ responses were based on a sound understanding of the instructions, the items, and the response format, participants were screened for their capacity to read and comprehend English at a high level. We examined the participants’ questionnaire responses, and responses that suggested a lack of comprehension were not included in the analyses cited in this paper. Based on standard questionnaire-checking processes, any participants whose responses showed signs of such response patterns were eliminated from the sample before the analyses were conducted. A small number of questionnaires were eliminated.

## The first stage of the study

### Item generation and content validity

An instrument referred to as the Asian Perceptions of Quality Physical Education (APQPE) was developed for this study based on the reviewed literature of Ho et al. (2017, 2018, 2019), Song and Chen (2012), Chen (2016), Keating and Silverman (2004), Guan, et al. (2005), Subramaniam and Silverman (2007) and Arar and Rigbi (2009) [15, 23–30] who have used the same methodology; this instrument was verified using a content validity procedure suggested by Lynn (1986) [31]. Existing instruments were not considered because they tended to be constructed within a specific cultural environment and setting, which may create idiosyncratic problems as a result of the formulation of items related to the specific culture [32]. To develop the questionnaire, the research group used references from the Quality Physical Education Guidelines developed by the National Association for Sport and Physical Education in 2004, the 2005 UNESCO report on Quality Physical Education, the ICSSPE 2010 International Position Statement on Physical Education and the preliminary works of the ICSP in 2010 on the development of International Benchmarks for Physical Education Systems. In addition to the above procedure, the authors of this study were also requested to provide valuable input based on empirical (observation or experience) and epistemological (theory of knowledge, logical evidence, etc.) approaches during item accumulation and construction. For instance, participating authors were acknowledged about the status (time allocation, lesson plan, administrative support etc.) of physical education in their own cities/countries, observed and experienced about the implementation of government policies into real practice including theoretical understanding and logically analysis the implementation of policies in terms of real benefits to the society at large etc.

Furthermore, the authors reached a clear consensus on checking the face validity, i.e., how closely the set of items aligned with real situations in the context of the Quality Physical Education in schools in their respective countries. Moreover, the authors’ input in the entire process was immensely productive, as all of them are well-established researchers in their respective countries and belong to the international streams of physical education, sports pedagogy and sports psychology; they are also quite concerned about the current policies and physical education provision prevalent in their countries [24]. Hence, the current instruments will be highly attuned to a specific cultural environment and setting, which may reduce idiosyncratic problems as a result of the formulation of items related to a specific culture [33].

The content validity of the professional perceptions of Quality Physical Education in this study was evaluated to determine whether all important aspects were covered, identified or essential, as well as to exclude items undesirable to a specific construct domain [15, 23–24]. The two-stage process for content validity developed by Lynn (1986) [32] was adopted. This two-stage process included developmental and judgement stages.

### The developmental stage

The first stage focused on defining the professional perceptions of Quality Physical Education (QPE), generating content domains in each component, and developing an item pool for each domain. Two methods were employed to generate the content domains and relevant items. The first method requires pooling relevant items from previous studies on the topic and subsequently generating new items. The second method is initiated by gathering items and domains from the target respondents. The advantage of employing both methods to generate content domains and the items in each domain is that it ensures that all relevant items and possible content domains are considered at the initiation of the instrument development [24–25, 27]. All items in the questionnaire were descriptive statements, and thus, the items were extensively reviewed by the authors. Additionally, the items were more focused based on the literature, and the authors subsequently related them to the context of their own respective countries.

This process resulted in the initial dimensions proposed, i.e., the status of physical education, the physical education curriculum in schools, physical education teachers and their qualifications, the infrastructure required to conduct physical education, teaching in physical education, the benefits of physical education, and the current challenges for physical education. The items from the literature reviews were subsequently generated to enable the assessment of each of the seven content domains. The authors identified 24 items regarding professional perceptions of Quality Physical Education (QPE). The items generated were also examined in terms of their clarity and readability. Twenty-four items were agreed on, and the items recommended by the authors represented the content validity. As a secondary process, six volunteer students *(who were familiar with the concept of Quality Physical Education in school settings)* from the University of Macau were asked to ascertain whether the items generated by the authors in each statement were sufficiently clear and relevant to describe professional perceptions of Quality Physical Education (QPE), verify whether important aspects or domains had been omitted, or whether a statement should be excluded from the existing items. The six students included one PhD student, two final-year master’s students, two sophomores, and one freshman [27, 24]. Three of the students studied physical education, and three students were in the social science field. According to their recommendations, four statements were revised. Thus, 24 items were maintained.

### The judgement stage

The judgement stage focused on the item validity and domain validity. Three external experts (physical education professors other than the authors) from other universities and the six previously described student participants were invited to participate in this judging process. The three professionals were invited to determine the face validity and to indicate whether the questionnaire provided an appropriate description regarding the study purpose and content area. The team also evaluated the questionnaire in terms of feasibility, readability, consistency of style, formatting, the clarity of the language used and domain validity [15, 23–24]. The adoption of these procedures was introduced by Haladyna (1999), Trochim (2001), DeVon et al. (2007) and DeVellis (2003) [34–37]. A quantitative sorting process was conducted to determine whether the statements fit the instrument in the assessment of professional perceptions of Quality Physical Education in school settings (QPE) and whether the statements were consistent with the seven corresponding dimensions. The participants were asked to indicate whether the statement should be included on a 3-point scale, with 1 = *No*, 2 = *Maybe*, and 3 = *Yes*, as well as how confident they were regarding the inclusion of an item (i.e., *1 = Not very sure*, 2 = *Sure*, and *3 = Very sure*) [24, 38–39]. A minimum of two of the three judges had to agree that a statement belonged to the instrument (where 3 = *yes*), and the mean confidence score was required to be greater than 2.0 (where 2 > *sure*). The judges were also asked to associate each of the 24 items with one of the seven dimensions and to indicate how confident they were that their selection was related to the specific content domain. The rating scales and criteria for domain validity were the same as the item validity criteria [40–41]. As a result, two items were revised, and one of the items was moved to a different content domain. Thus, 24 items were maintained in the instrument and classified into the seven original dimensions. The six volunteer students were subsequently invited to verify the item validity and domain validity based on the experts’ classification [23–24]. The same procedures and regulations were adopted. As a result, no modifications were required for the items.

The QPE questionnaire comprised two sections. The first section contained the 24 items regarding the QPE, and the participants were asked to indicate how strongly they agreed with each statement with regard to Quality Physical Education in the schools in their respective countries.

They were asked to respond on a 6-point, positively packed, agreement-rating scale. This response scale included two negative and four positive agreement responses with identical scores (i.e., strongly disagree = 1, mostly disagree = 2, slightly disagree = 3, moderately agree = 4, mostly agree = 5, and strongly agree = 6). Positively packed agreement-rating scales are known to generate discrimination in the context of social desirability [24, 38–39].

Studies have shown that people responding to self-reported questionnaires on sensitive topics often undermine their accuracy or respond in ways that can produce a multitude of errors in order to present themselves in the best possible light; this phenomenon is referred to as social desirability bias [40–41]. Therefore, investigators must understand procedures to prevent or alleviate this reported bias in the design of their questionnaires [42], often through the questionnaire’s psychometric properties [43]. Apparently, five- to seven-point rating scales are quite popular choices by investigators for use in questionnaires. However, in order to obtain finer discriminations within a portion of the rating-scale continuum, McKelvie (1978) and Comrey (1978) [44] have suggested that so-called neutral midpoints should be avoided in a rating scale. The present study is based on agreement formats, as Schwarz (1999) [45] identified that self-report inventories perform better if agreement rather than frequency response formats are used. Agreement formats may be less prone to memory error since they elicit information based on the present rather than asking for recalled knowledge, beliefs, opinions, or attitudes of respondents [38, 45]. Therefore, because positively packed rating scales, i.e., more response options representing the positive end of the continuum, generated lower bias [39], a six-point rating scale is used in the present study in the hopes that participants would attend to the meaning of the intermediate response options.

## The second stage of the study

The second section comprised the participants’ personal demographic information.

## Results

Of the total dataset, only .41% was missing cases, and 99.59% of the available data were subjected to statistical analysis. This procedure followed the description suggested by Dempster, Laird and Rubin (1977) [46] regarding missing values at 5%. The data were verified and deemed acceptable for further analysis. Both statistical and empirical techniques were used to select the items. Twenty-four items were subjected to descriptive and frequency analyses (Table 3). Using SPSS 20.00 (IBM), the research team examined the data quality in terms of its frequency distribution and item discrimination. An exploratory factor analysis (EFA) with maximum likelihood extraction and direct oblimin rotation was adopted to investigate the structure of Quality Physical Education and define a set of factors that accounted for the common variance among the items. These items were subsequently evaluated by their loading on each factor. The second phase of the analysis was conducted to confirm the different subscales and the structure of the 24 items. A reliability analysis (Cronbach’s alpha) was performed to determine the contribution of each item to its respective factor. When items were deemed to be statistically equivalent, the authors were asked to determine which items to retain and place within the appropriate categories to reflect their close conceptual meaning.

**Table 3:** Descriptive statistics on all the retained items.

Items No.	Descriptions of the items	Mean	SD	Skew.	Kurt.
**Item** 2	Physical Education should be accessible to all children, whatever their ability/disability, sex, age, culture, race/ethnicity, religious, social or economic background.	5.54	.892	-2.61	8.01
**Item** 3	Physical education should be a compulsory subject in school for all children	5.67	.799	-3.40	13.79
**Item** 4	The school should have safe and suitable equipment for physical education lessons	5.70	.759	-3.89	18.74
**Item** 5	The school should have safe and suitable facilities for physical education lesson	5.69	.744	-3.57	15.60
**Item** 6	The School should have safe and suitable environment for physical education lessons	5.69	.715	-3.53	16.39
**Item** 7	The Teacher should be qualified to teach physical education	5.32	1.09	-2.02	4.11
**Item** 8	Different types of physical activities and associated knowledge should form the content through which young people learn	5.19	1.09	-1.65	2.67
**Item** 10	Positive sport related attitudes and values should form a major focus on learning	5.35	.919	-1.75	3.89
**Item** 11	The teaching and learning of physical education should be fun and enjoyable	5.39	.934	-2.04	5.21
**Item** 12	Students should be given opportunities for active learning in physical education lesson	5.48	.832	-2.38	7.87
**Item** 13	Extension physical activity opportunities, after-school or extra-curricular / co-curricular activities are essential components in helping students to extend their learning experiences in sport and physical activities	5.22	.996	-1.67	3.54
**Item** 15	All schools have safe and suitable equipment for physical education lessons	3.93	1.11	.080	-.500
**Item** 16	All schools have safe and suitable facilities for physical education lessons	3.83	1.11	.069	-.300
**Item** 17	All schools have safe and suitable environment for physical education lessons	3.81	1.13	.178	-.410
**Item** 19	Different types of physical activities and associated knowledge form the major content in learning	4.28	1.03	-.200	-.426
**Item** 20	Health knowledge is regarded as the major content in learning	4.20	1.25	-.441	-.382
**Item** 21	Positive sport related attitudes and values are taught and form the major content in learning	4.42	1.17	-.621	.102
**Item** 22	The teaching and learning of physical education is fun and Enjoyable	4.45	1.03	-.267	-.257
**Item** 23	Students are given opportunities for active learning in physical education lessons	4.28	1.07	-.226	-.326
**Item** 24	Extension physical activity opportunities, after-school or extra-curricular / co-curricular activities are available to all students to extend their learning experiences in sport and physical activities	4.20	1.17	-.318	-.627

Furthermore, confirmatory factor analysis (CFA) using AMOS 21 (IBM) was conducted to examine the retained three-factor structure from exploratory factor analysis. The overall model fit was evaluated using multiple goodness-of-fit indices including the Chi-square value, comparative fit index (CFI), the Tucker-Lewis Index (TLI), parsimonious comparative fit index (PCFI), and the root mean square error of approximation (RMSEA) accompanied by its 90% confidence interval (90% CI). Although much debate surrounds the selection of precise thresholds of fit, especially relevant within the field of theory-based multi–item/factor CFA testing [47], it is commonly accepted that thresholds of >.90, close to (or less than) .08 and up to .08 [48–49] for the CFI and RMSEA are indicative of acceptable model fit [47, 50, 24].

### Phase I

**Fig 1:**
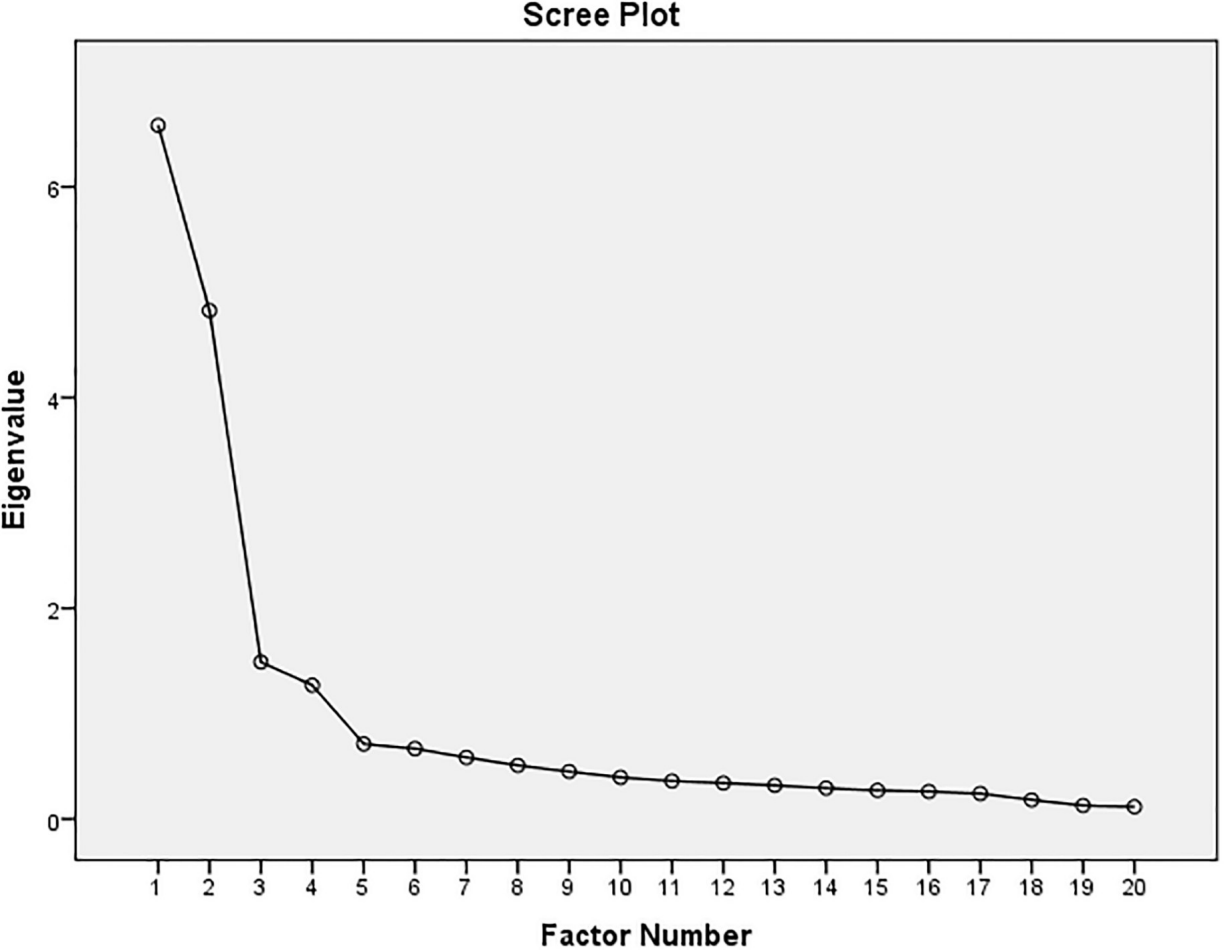
Scree Plot depiction based on the eigenvalues.

The major concerns of the present study were to identify a potential framework for the investigation of Quality Physical Education and subsequently determine a structure for analysis. To achieve the primary purpose of the study, i.e., to define a set of factors that would account for Quality Physical Education, the results of a maximum likelihood extraction with direct oblimin rotation are presented (Table 4). To determine the number of factors, several criteria, including the differences between the adjacent eigenvalues, a scree plot and the differences in the percentage of variance were used to account for the adjacent factors and, more importantly, to consider the factor structure (Fig 1).

**Table 4:** Factor loadings based on pattern matrix and communalities (h2) retained after exploratory factor analysis.

Items No.	Descriptions of the Factors	Factors Loading				*h*^*2*^
		1	2	3	4	
**Development of Supportive Elements for Quality Physical Education in School (DSFQPE)**						
**Item** 16	All schools have safe and suitable facilities for physical education lessons	.979				.*955*
**Item** 15	All schools have safe and suitable equipment for physical education lessons	.869				.*762*
**Item** 17	All schools have safe and suitable environment for physical education lessons	.819				.*685*
**Core Value of Quality Physical Education (CVPE)**						
**Item** 4	The school should have safe and suitable equipment for physical education lessons		.927			.*809*
**Item** 5	The school should have safe and suitable facilities for physical education lesson		.915			.*852*
**Item** 6	The School should have safe and suitable environment for physical education lessons		.901			.*781*
**Item** 3	Physical education should be a compulsory subject in school for all children		.663			.*570*
**Item** 11	The teaching and learning of physical education should be fun and enjoyable		.596			.*382*
**Item** 2	Physical Education should be accessible to all children, whatever their ability/disability, sex, age, culture, race/ethnicity, religious, social or economic background.		.582			.*401*
**Item** 12	Students should be given opportunities for active learning in physical education lesson		.556			.*556*
**Curriculum Arrangement of Physical Activities (CAPA)**						
**Item** 21	Positive sport related attitudes and values are taught and form the major content in learning			.926		.*778*
**Item** 20	Health knowledge is regarded as the major content in learning			.747		.*577*
**Item** 22	The teaching and learning of physical education is fun and Enjoyable			.738		.*554*
**Item** 23	Students are given opportunities for active learning in physical education lessons			.712		.*544*
**Item** 19	Different types of physical activities and associated knowledge form the major content in learning			.656		.*577*
**Item** 24	Extension physical activity opportunities, after-school or extra-curricular / co-curricular activities are available to all students to extend their learning experiences in sport and physical activities			.626		.*490*
**Provision and Norms in Physical Education (*PNPE)***						
**Item** 8	Different types of physical activities and associated knowledge should form the content through which young people learn				.851	.*721*
**Item** 10	Positive sport related attitudes and values should form a major focus on learning				.733	.*686*
**Item** 7	The Teacher should be qualified to teach physical education				.719	.*598*
**Item** 13	Extension physical activity opportunities, after-school or extra-curricular / co-curricular activities are essential components in helping students to extend their learning experiences in sport and physical activities				.503	.*542*

*Note*: Extraction Method: Maximum Likelihood. Rotation Method: Oblimin with Kaiser Normalization.

a. Rotation converged in 5 iterations. (N = 399).

A solution with four factors (subscales) was presented. Factor One was referred to as the “Development of Supportive Elements for Quality Physical Education in School (DSEQPE)”, Factor Two was referred to as the “Core Values of Quality Physical Education (CVQPE)”, Factor Three was referred to as the “Curriculum Arrangement of Physical Activities (CAPA)” and Factor Four was referred to as the “Provision and Norms in Physical Education (PNPE)”. These factors had eigenvalues of 6.758%, 4.825%, and 1.490%, and 1.27% respectively, which explained 1.270% of the variance.

As shown, the Asian professionals differentially perceived the development of supportive elements for Quality Physical Education in schools, core values (such as safety and accessibility) and curriculum arrangement. Nevertheless, the internal consistency (Cronbach’s alpha coefficient) for the three subscales was calculated. Based on the item statistics, seven items from the Core Values of Quality Physical Education (CVPE), three items from the Development of Supportive Elements for Quality Physical Education in School (DSFQPE), six items from the Curriculum Arrangement of Physical Activities (CAPA) and four items from the Provision and Norms in Physical Education (PNPE) were selected and retained because of their good internal consistencies. Of the original set of 24 items, four items with low factor loadings were excluded from the analysis; thus, the list of the remaining items trimmed to 20 only.

## Phase II: Underlying structure of the professional perception toward Quality Physical Education (PPQPE)

**Fig 2:**
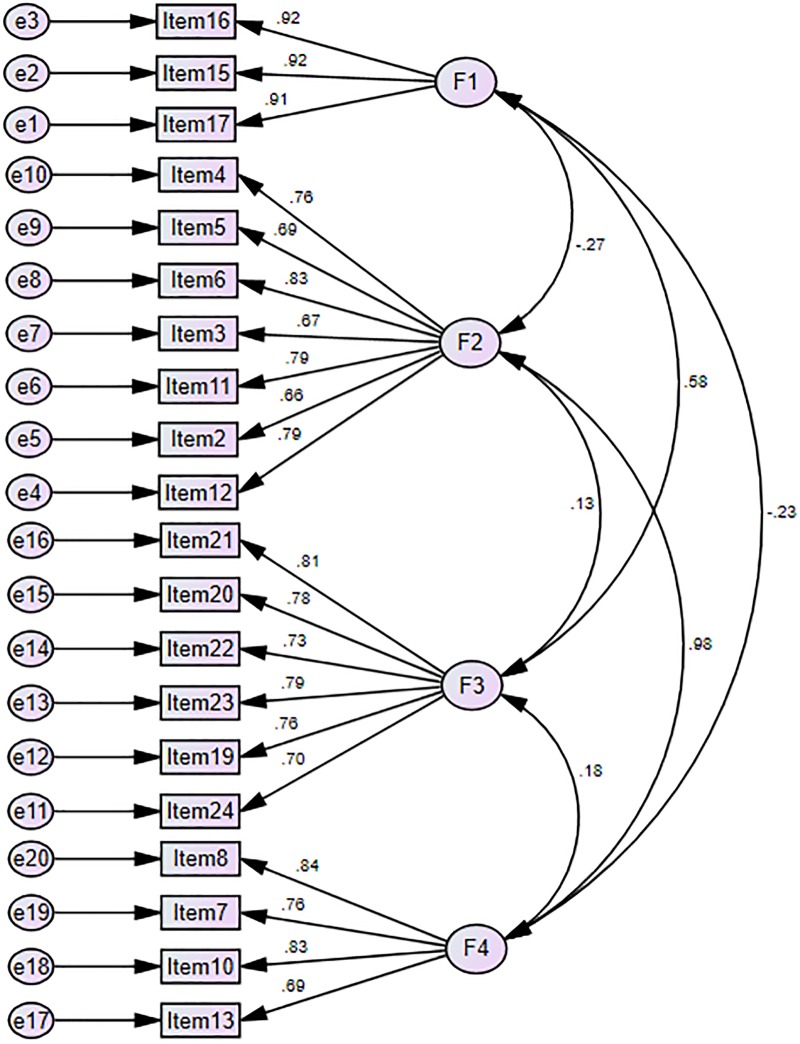
Measurement model for QPE. Note: Factor 1- Core Values of Quality Physical Education (CVPE), Factor 2- Development of Supportive Elements for Quality Physical Education in School (DSFQPE), Factor 3- Provision and Norms in Physical Education (CAPA), and Factor 4- Provision and Norms in Physical Education (PNPE).

The results of the factor analysis indicated that the 20 items listed in the final version of the questionnaire demonstrated sound and good inter-correlation results, as evidenced by the high value (.895) of the Kaiser-Meyer-Olkin measure of sampling adequacy (MSA) and a significant Bartlett’s test of sphericity. The MSA comprised an index used to quantify the degree of inter-correlation among the items and the appropriateness of the factor analysis [23, 25]. A measure that calculated a value greater than .50 for the entire matrix or an individual variable would indicate the appropriateness of acceptance [51]. Further, all the items with factor loadings greater than .50 were retained. When the pattern matrix *(factor and structure matrix were considered because of cross-loading)* was considered, three subscales were retained to reflect the conceptual framework [23–24]. These three basic subscales included the Core Values of Quality Physical Education (CVPE), Development of Supportive Elements for Quality Physical Education in School (DSFQPE), Curriculum Arrangement of Physical Activities (CAPA) and Provision and Norms in Physical Education (PNPE) (Fig 2).

### Internal consistency of the professional perception of Quality Physical Education

The internal consistency reliability coefficients (Cronbach’s alpha) for each subscale were computed. As shown in Table 5, the Cronbach’s alpha coefficient was .918 for the Development of Supportive Elements for Quality Physical Education in School (DSFQPE) scale. For the Core Values of Quality Physical Education (CVPE) factor, the value was .908. Moreover, the sub-factor of the Curriculum Arrangement of Physical Activities (CAPA) was .884. The last sub-factor of the Provision and Norms in Physical Education (PNPE) was .865. These values indicated that the items were consistent within each factor and that the factors were consistent within the model to permit meaningful further analysis. The inter-correlations between the four major practices were moderate and ranged from -.037–0.627. The factors Development of Supportive Elements for Quality Physical Education in School (DSEQPE), Core Values of Quality Physical Education (CVPE) and Provision and Norms in Physical Education (PNPE), and Curriculum Arrangement of Physical Activities (CAPA) were strongly correlated, whereas the factors Core Values of Quality Physical Education (CVPE), Curriculum Arrangement of Physical Activities (CAPA), and Provision and Norms in Physical Education (PNPE) were moderately correlated.

**Table 5:** Inter-factor correlation, Cronbach’s alpha and descriptive statistics for QPE.

Factor	1 (DSFQPE)	2 (CVPE)	3 (CAPA)	4 (PNPE)	α	Mean	SD	Variance	*No*. *of Items*
1(DSFQPE)	1.000	-.037	.528	.018	.918	11.59	3.12	9.76	3
2 (CVPE)		1.000	.114	.627	.908	39.19	4.57	20.95	7
3 (CAPA)			1.000	.127	.884	25.88	5.36	28.79	6
4 (PNPE)				1.000	.865	21.13	3.45	11.95	4

*Note*: 1- Development of Supportive Elements for Quality Physical Education in School (DSFQPE), 2- Core Value of Quality Physical Education (CVPE), 3- Provision and Norms in Physical Education (CAPA), and 4- Provision and Norms in Physical Education (PNPE)

Descriptive results regarding the factor mean scores were calculated. In general, the professionals reported the most positive attitudes towards the Core Values of Quality Physical Education (CVPE) (M = 39.19; SD = 4.57), followed by the Curriculum Arrangement of Physical Activities (CAPA) (M = 25.88; SD = 5.36). The lowest mean was identified for the Development of Supportive Elements for Quality Physical Education in School (DSEQPE) (M = 11.59; SD = 3.12), followed by the Provision and Norms in Physical Education (PNPE) (M = 21.13; SD = 3.45).

To check the retained factors’ item loading, a measurement model was evaluated using multiple goodness-of-fit indices, including Chi-square value, CFI, TLI, PCFI, and RMSEA accompanied by 90% confidence intervals (90% CIs) (Table 6). The results of the robust CFA, using the maximum likelihood estimation method (see Table 5), suggest that the three-factor model provided an adequate model fit to the data [23–24].

**Table 6:** Model fit indices for the data collected using QPE.

	Model H0
N	400
CMIN	565.773
DF	164
CMIN/DF	3.450
CFI	.928
TLI	.916
GFI	.875
PCFI	.801
RMSEA	.078

Model H0 = the hypothesized model. N = sample size. CMIN = minimum discrepancy. DF = degrees of freedom. CFI = comparative fit index. TLI = Tucker Lewis index, PCFI = parsimony comparative fit index, RMSEA = root mean square error of approximation.

## Discussion of findings

In this study, twenty-four items were listed in the questionnaire, and twenty items were extracted following an exploratory factor analysis. Item no. 1 (Physical Education is the most effective means of equipping children with the needed skills, attitudes, values, knowledge), 9 (Health knowledge should be regarded as one of the major areas of learning), 14 (Physical education should be a compulsory subject in school) and 18 (All teachers are qualified to teach physical education) were not retained because of the low factor loadings.

All statements in the Development of Supportive Elements for Quality Physical Education in School (DSEQPE) factor exhibited a Mean ± SD of 11.59 ± 3.12. Nevertheless, the factor earned a reliability score of α = .918 as calculated with Cronbach’s alpha, which included items such as ‘all schools should have safe and suitable facilities, equipment, and an environment for physical education lessons’ and ‘all teachers are qualified to teach physical education’. The responses from professionals indicated hesitation regarding these statements. These findings were consistent with the study by Hardman (2009) [52], which indicated that physical education commonly faces the challenges of inadequate facilities and poor maintenance of teaching sites. Thus, these challenges comprise essential factors in the effective development of physical education. The findings of this factor indicate a source of worry, as facilities, venues/settings and use of equipment comprise the image of physical education. Suggestions for further discussion are needed with the goal to determine whether professionals who are accustomed to poor facilities have perceptions that lead to the negative adaptation of the environment and reduce hope for the quality improvement of physical education. Negative feelings should be avoided to provide positive incentives for physical education professionals to remain in the job with hope and positive prospects for the future.

Furthermore, the findings of this study provided sources in different dimensions to describe the works of Quality Physical Education and the framework for discussion. For example, the Core Values of Physical Education (CVPE) factor included items regarding safe and suitable environments for physical education, making physical education a compulsory subject, accessibility of PE without discrimination or barriers, fun and enjoyment in learning, opportunities for active participation, and suitable sport-related educational content. The factor exhibited a Mean ± SD of 39.19 ± 4.57, as well as an sufficient reliability (α = .908). These factors exhibited the required reliability; thus, it is expected that professionals would consider them important for the establishment of Quality Physical Education in schools. The “core values” may be best viewed as attributes of Quality Physical Education. These attributes were related to opportunities for physical activity, educational content, the development of health-related fitness, the establishment of physical competence and other educational growth areas, such as critical thinking, creativity, collaborative skills and cognitive understanding [53, 23–24]. The attributes selected by the professionals matched the holistic concepts indicated by the National Association of Sport and Physical Education (2004) [3**]** regarding curriculum, content knowledge, instruction and assessment; the factors discussed by Whitehead (2001) [54**]** regarding physical literacy in motivation, confidence, physical competence, knowledge and understanding; the factors indicated by Kumar (2017) [55] regarding social value and significance in individuals; and the factors suggested by Mottet and Beebe (2006) [56] regarding the affective domain in attitudes, beliefs and value development.

The Curriculum Arrangement of Physical Activities (CAPA) factor exhibited a Mean ± SD of 25.88 ± 5.36. This factor also had high reliability (α = .884). A high mean and high reliability indicated the utmost importance of this factor in the realm of Quality Physical Education by professionals. The factor was best described as “inevitable essential qualities” for proper curriculum arrangement and plays a significant role in the development of learning motives, goal achievement and habit development in the participation of sport and physical activities. The items included positive attitudes and values, content knowledge, learning quality, opportunities for active learning and the possibility of extension of learning experiences and emphasis on after-school physical activities. In connection with the findings of the present study, the research of Eccles and Gootman (2002) [57] may also indicate the relationship of habits in sport and physical activities, opportunities for participation in school and community-based activities and the importance of both short-term and long-term indicators in the positive development of sport-related attitudes and values in students. Studies have shown that engagement in physical activity, support in developmental activities and engagement in challenging tasks enabled participants to express their talents, passion, and creativity, thereby easily enhancing the development of habits of active participation [53, 58–59].

The fourth of the sub-factors extracted by the EFA was referred to as the Provision and Norms in Physical Education (PNPE). The factor exhibited a Mean ± SD of 21.13 ± 3.45. This factor also had high reliability (α = .861). This subscale consists of 4 items to indicate professionals’ perspective about types of physical activities and associated knowledge in physical education, positive sport-related attitudes and values, importance of qualified teachers, and extension of opportunities for enhancing learning experiences in physical education. UNESCO’s International Charter of Physical Education and Sport (Article 5) in 2015 recommended that adequate provisions such as safe spaces, facilities, equipment, and dress options must be provided to all children in physical education and sports, while being mindful of different needs associated with climate, culture, gender, age, and disability. In the context of maintaining the norms of the profession, the professionals expressed the view that the principle of free physical education should be upheld for all children, irrespective of their ability/disability, sex, age, culture, race/ethnicity, and religious, social or economic background. Fun activities, environment building, opportunities for activities, a useful arrangement for sport-related curriculum, content knowledge, and positive instruction and assessment were identified as the major ingredients for a Quality Physical Education [3].

In addition, the results from the CFA on the 20 QPE items revealed a desirable goodness of fit between the proposed 4-factor model and the data collected from this substantial sample of participants in diverse types of PE in the context of large cities in Asia. Furthermore, the high, unmediated effects of the latent variables on the observed variables indicated that the items are actually measuring what they have been assigned to measure. Hence, the results reported here suggest that the hypothesized model in the current study fitted the data well, lending support to the initial validity of the QPE. The present results support the applicability of this scale as a measure of a wide range of Quality Physical Education characteristics among professionals in diverse PE contexts [23–24].

## Conclusion

An overarching concern for Quality Physical Education has been the lack of reliable and valid measures of Quality Physical Education in schools. In recent years, research has been conducted to discuss the issue of Quality Physical Education; however, there has been a lack of suitable answers that may best predict the basic elements in the construction of Quality Physical Education and sport programmes for students. The items in this study exhibited high consistency and were regarded by professionals who originated from different backgrounds as essential criteria for the investigation of Quality Physical Education in Asia. These items reflect characteristics regarding the role, overall functions and arrangement of physical education in school settings; the development of educational elements, such as curriculum, instruction, and the internal quality of physical education lessons and after school programmes; and the establishment of supportive features, such as venues, facilities, equipment and environments. The items indicated in the three factors helped to envisage the development of a basic framework for the analytical work of Quality Physical Education in Asian schools and reflected the perception of the basic issues in the development of Quality Physical Education programmes. Nevertheless, these observations require further investigation because of the lack of comparative data. This study was conducted in 11 Asian cities, and many metropolitan cities, such as Tokyo and Beijing, and countries were not included. As a result of the limited sample size, the observations cannot be applied or generalized as common phenomena for Quality Physical Education in Asia. Nevertheless, this study highlights the concerns regarding and the approaches to constructing Quality Physical Education in schools.
